# UAV-Assisted Mobile Edge Computing: Dynamic Trajectory Design and Resource Allocation

**DOI:** 10.3390/s24123948

**Published:** 2024-06-18

**Authors:** Zhuwei Wang, Wenjing Zhao, Pengyu Hu, Xige Zhang, Lihan Liu, Chao Fang, Yanhua Sun

**Affiliations:** 1 Faculty of Information Technology, Beijing University of Technology, Beijing 100124, China; wangzhuwei@bjut.edu.cn (Z.W.); 836118497@emails.bjut.edu.cn (W.Z.); fangchao@bjut.edu.cn (C.F.); sunyanhua@bjut.edu.cn (Y.S.); 2 Department of Smart Agriculture Engineering, Shanghai Vocational College of Agriculture and Forestry, Shanghai 201699, China; 3 Beijing Institute of Astronautical Systems Engineering, Beijing 100076, China; zhangxg01casc@163.com; 4 School of Statistics and Data Science, Beijing Wuzi University, Beijing 101149, China; liulihan@bwu.edu.cn

**Keywords:** UAV, MEC, trajectory design, task offloading, resource allocation

## Abstract

The recent advancements of mobile edge computing (MEC) technologies and unmanned aerial vehicles (UAVs) have provided resilient and flexible computation services for ground users beyond the coverage of terrestrial service. In this paper, we focus on a UAV-assisted MEC system in which the UAV equipped with MEC servers is used to assist user devices in computing their tasks. To minimize the weighted average energy consumption and delay in the UAV-assisted MEC system, a LQR-Lagrange-based DDPG (LLDDPG) algorithm, which jointly optimizes the user task offloading and the UAV trajectory design, is proposed. To be specific, the LLDDPG algorithm consists of three subproblems. The DDPG algorithm is used to address the issue of UAV desired trajectory planning, and subsequently, the LQR-based algorithm is employed to achieve the real-time tracking control of UAV desired trajectory. Finally, the Lagrange duality method is proposed to solve the optimization problem of computational resource allocation. Simulation results indicate that the proposed LLDDPG algorithm can effectively improve the system resource management and realize the real-time UAV trajectory design.

## 1. Introduction

The rapid development of mobile intelligent devices is boosting the growth of the Internet of Things (IoT) and the advent of complex mobile applications with intelligent features, such as face recognition, video processing, and online games [1]. These applications are typically latency- and computation-sensitive. However, IoT devices, due to their relatively low computing and battery capabilities, are unable to maintain superior performance [2]. Although cloud computing can offload terminal computing tasks to cloud servers, thereby alleviating the computational burden on mobile devices, the task offloading will cause excessive latency and link congestion problems [3,4].

Mobile edge computing (MEC) provides a cost-efficient solution for computationally intensive and latency-critical tasks, by allocating computational resources towards the network edge to users [5]. The edge execution of user tasks extends the battery life of devices, reduces the power consumption and latency associated with communication and local computing, and improves the quality of service [6,7]. However, in traditional MEC application scenarios, the communication links are dominated by non-line-of-sight (NLoS), which indicates that the data transmission rate is severely restricted by the poor quality of the communication channel [8]. In addition, it poses a significant challenge in deploying the terrestrial MEC unit in certain situations, such as in remote areas or during emergency events [9].

Fortunately, the technology of unmanned aerial vehicles (UAVs), characterized by flexible mobility, easy deployment, and line-of-sight (LoS) connections, has gradually become an important component of future wireless networks. The UAV-assisted MEC system provides a potential solution to address the aforementioned challenges in terrestrial MEC systems [10,11]. Compared to traditional wireless networks, UAV-assisted MEC networks offer a multitude of advantages in terms of mobility, flexibility, cost, coverage, and reconfiguration. Moreover, UAVs equipped with MEC servers can approach users closely to provide services, which can notably reduce energy consumption and transmission delay.

However, designing a joint optimal scheme for resource management and UAV trajectory planning faces significant challenges due to the UAV’s inherent dynamics constraints and limited onboard computation capability and energy resources [12,13,14]. On the one hand, the actual UAV flight acceleration and velocity cannot be adjusted arbitrarily, so sudden acceleration, deceleration, and turning are impossible. However, they are often completely overlooked in existing UAV trajectory planning, resulting in significant deviations between the actual flight trajectory and the theoretically designed trajectory of the UAV [15]. On the other hand, UAV trajectory planning requires achieving coverage for all users and satisfying the task offloading requirements. However, both the users’ task offloading ratio and the communication channel between the user and the UAV are time-varying, which causes performance degradation on offloading efficiency, latency, and energy efficiency. Therefore, given the high dynamic scenarios and the frequent task offloading requirements of users, the resource management of MEC systems and the design of UAV flight trajectories have become crucial research topics.

Motivated by the above-mentioned reasons, this paper focuses on the UAV-assisted MEC system considering UAV flight dynamics constraints. A novel linear quadratic regulator (LQR)-Lagrange-based deep deterministic policy gradient (LLDDPG) algorithm is proposed to minimize the weighted energy consumption and delays of the system through the joint optimization of dynamic computation resources and UAV flight trajectory. In fact, in light of the UAV flight dynamics restriction that the velocity and acceleration of the UAV cannot change arbitrarily, the UAV is required to replan a feasible flight trajectory based on the UAV’s current flight state and the task offloading requirements of users, thereby enhancing the system performance. The main contributions of this work are summarized as follows:

- Taking into account the dynamic control of the UAV trajectory, the system architecture for a UAV-assisted MEC is investigated. The communication model, UAV control model, as well as the computing and transmission model are analyzed in detail. Subsequently, the joint optimization problem minimizing the weighted energy consumption and delay is formulated when considering the UAV dynamics constraint.
- Constrained by the system dynamics of the UAV, where the velocity and acceleration are not allowed to change arbitrarily, a LLDDPG algorithm is proposed to address the joint dynamic trajectory and resource allocation problem. Specifically, for a practical solution, the optimization problem is decomposed into three distinct subproblems. Firstly, a DDPG-based UAV trajectory design algorithm is developed to acquire the desired optimal trajectory. Subsequently, the LQR-based tracking control algorithm is introduced to derive the actual UAV flight trajectory subject to the system dynamics. Finally, the resource allocation problem regarding the offloading ratio and computation frequency assignments is solved using the Lagrange duality method.
- Numerical simulation results extensively demonstrate the efficacy of the proposed LLDDPG algorithm in terms of learning rate, loss function, and reward convergence. Additionally, the performance evaluations with different weight parameters and the effectiveness of the LLDDPG algorithm in actual UAV flight control are also investigated and analyzed.

The remainder of the paper is organized as follows. In Section 2, the related works are reviewed. The UAV-assisted MEC system model and the optimization problem formulation are presented in Section 3. In Section 4, the LLDDPG algorithm is proposed. In Section 5, numerical simulations and results are presented. Finally, conclusions are drawn in Section 6.

## 2. Related Works

This section briefly reviews the works related to UAV-assisted MEC resource allocation and trajectory design, and the existing issues and challenges are also discussed.

### 2.1. UAV-Assisted MEC Resource Allocation

In recent years, the increasing maturity of UAV-assisted wireless communication technology has boosted the further development of MEC systems. How to combine UAV advantages and MEC networks has become a research hotspot [16,17,18,19,20,21,22,23,24,25]. Guo et al. [16] introduced a UAV-enabled MEC system, in which the UAV served as a relay between the base station and the offloading user. This work investigated the joint optimization of flight trajectories and computational offloading, considering both user service quality and energy consumption. In [17], it maximized the UAV’s transmit power efficiency by jointly optimizing bandwidth assignment, transmission time, UAV placement, and power allocation control. Furthermore, Qin et al. [18] investigated the energy efficiency of a UAV-assisted MEC system by considering energy consumption and the device task requirements.

The above works mainly focused on the energy efficiency of the MEC system, while ignoring the influence of the UAV trajectory design. In order to improve the network lifetime and computation capability associated with the UAV, Wang et al. [19] investigated an optimization problem that aimed to minimize the total energy consumption of the UAV through a combined approach of zone division and UAV trajectory planning. Wang et al. [20] addressed the efficiency maximization problem by jointly optimizing bandwidth management, UAV trajectory, computation offloading, and computation resource assignment. Diao et al. [21] optimized the computational offload strategy and UAV trajectory in the UAV-enabled MEC system. Their objective was to minimize the total energy consumption and delay while enhancing the user’s service quality. Hu et al. [22] also focused on the joint optimization problem to maximize the data offloading efficiency while minimizing the UAV energy consumption. Liu et al. [23] put forward a system energy minimization problem subject to constraints such as UAV trajectory, transmit power, and CPU frequency. Zeng et al. [24] investigated the problem of minimizing UAV energy consumption, including propulsion energy and communication-related energy, while satisfying the communication throughput requirements of each ground node. By leveraging the traveling salesman problem (TSP) with neighborhood and convex optimization techniques, a successive convex approximation (SCA)-based algorithm is proposed. Yang et al. [25] considered a UAV-enabled MEC system to jointly optimize UAV energy and trajectory control while satisfying long-term data queue stability, and then a perturbed Lyapunov optimization-based offloading and trajectory (PLOT) control algorithm was proposed.

In the aforementioned research, the authors delved into the joint optimization of variables such as UAV trajectory, offloading strategy, computation frequency, and transmission power. Nevertheless, these existing works predominantly focus on the design of desired UAV flight trajectory, completely disregarding the restrictions imposed by UAV flight dynamics. In reality, the inherent limitations in the UAV’s flight capabilities can result in a growing deviation between the desired and actual flight trajectories, which might potentially lead to significant performance degradation.

### 2.2. UAV Trajectory Control

In order to ensure the efficiency of the UAV-assisted MEC system, it is crucial to jointly optimize the system resource allocation and UAV trajectory control. Since the UAV trajectory directly influences the MEC system’s energy consumption and user service quality, it is of great importance to track and control the flight trajectory. The trajectory flight control problem continues to attract significant attention due to its potential to enhance the system’s adaptability and its capability to handle dynamics and uncertainties [26,27,28,29,30]. Addressing the UAV trajectory tracking control problem, Yan et al. [26] proposed a dynamic tracking method for UAV landing trajectories based on chaos genetic algorithms. Lee et al. [27] proposed a trajectory tracking control methodology utilizing backward stepping and LQR control. Furthermore, Li et al. [28] presented a control-oriented UAV trajectory design approach that incorporates both the kinematics and dynamics equations of the UAV. However, these works primarily focused on the UAV’s trajectory tracking control and neglected the effect of tracking deviation on the overall performance of the UAV-assisted MEC system. Zhang et al. [29] tried to investigate this limitation by considering a network control system with delays, and an adaptive dynamic programming-based tracking control algorithm was proposed to generate real-time control actions. Liu et al. [30] focused on the UAV trajectory planning problem for an environmental monitoring system. The formulated optimization problem was divided into two subproblems: the UAV velocity optimization and trajectory optimization. To address these subproblems, the solving algorithms, based on SCA and general algorithm (GA), respectively, were proposed.

Regrettably, there have been few studies that take into account the joint design of system resource allocation and real-time UAV trajectory control in the UAV-assisted MEC system. Most of the existing articles have studied the desired trajectory planning, assuming that the UAV has the perfect flight capability and operates in a static transmission environment. In this paper, we aim to address this gap to investigate the inherent constraints of UAV flight dynamics, and focus on the joint optimization problem of dynamic trajectory design and resource allocation for a UAV-assisted MEC system.

## 3. System Modeling and Optimization Problem Formulation

**Figure 1:**
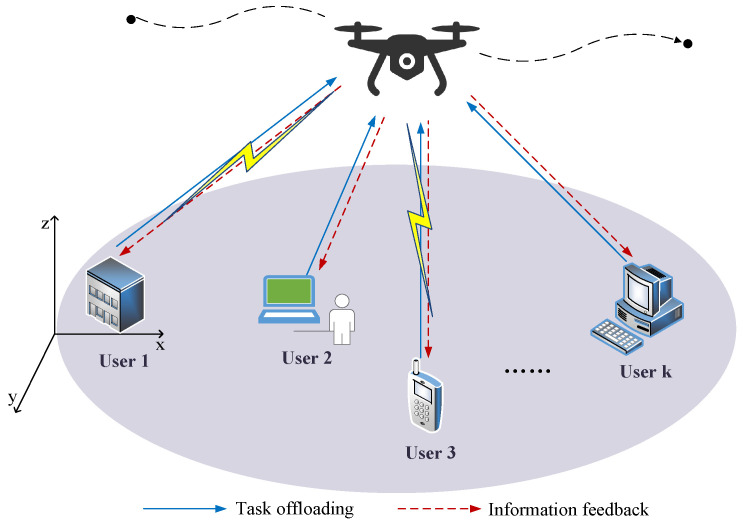
A UAV-assisted MEC system.

A UAV-assisted MEC system is depicted in Figure 1, which consists of the UAV and multiple users. The UAV, equipped with an MEC server, is capable of simultaneously transmitting information and providing edge computing service. Considering the limited computation capacities, the users are required to offload a portion of their computing tasks to the MEC server through the shared wireless network. Generally, the user’s computing tasks can be divided into two parts; one is computed locally, and the other is offloaded to the UAV for processing. The UAV aggregates the received information to form a new global model, and then feeds back the global information to the users. In order to enhance the energy efficiency and address the dynamic nature of entire system environment, the UAV’s flight needs to be frequently adjusted and controlled.

### 3.1. Communication Model

Let $q_{k}=\left\{x_{k},y_{k}\right\}$ denote the location of the *k*-th ground user, which is assumed to be known by the UAV. The position of the UAV in the *n*-th time slot can be represented as $q\left[n\right]=\left\{x_{u}\left[n\right],y_{u}\left[n\right],H\right\}$, where *H* is the fixed flight altitude of the UAV. Typically, it is assumed that the wireless channel between the user and the UAV is mainly dominated by the LoS. Thus, the channel gain $g_{k}\left[n\right]$ between the user *k* and the UAV in the *n*-th time slot can be expressed as [31]

(1) $$\begin{array}{c} g_{k}\left[n\right]=\beta_{0}d_{k}^{-2}\left[n\right] \end{array}$$

where $\beta_{0}$ is the channel coefficient and $d_{k}\left[n\right]$ is the distance between the user *k* and the UAV for which $d_{k}\left[n\right]\;=\;\sqrt{{\left(x_{u}\left[n\right]-x_{k}\right)}^{2}+{\left(y_{u}\left[n\right]-y_{k}\right)}^{2}+H^{2}}$.

Subsequently, the transmission data rate from the user *k* to the UAV can be derived as

(2) $$\begin{array}{c} R_{k}\left[n\right]=B\log_{2}\left(1+\frac{g_{k}\left[n\right]p}{N_{0}}\right) \end{array}$$

where $N_{0}$ is the noise power, and *B* and *p* represent the assigned bandwidth and transmit power, respectively.

Similarly, the transmission data rate from UAV to the user *k* is given by Ru[n]=Blog21+gk[n]pugk[n]puN0N0, where $p_{u}$ represents the transmit power of the UAV.

### 3.2. Computing and Transmission Model

Considering the partial offloading computation scenario, the computation tasks can be divided into two parts. One is handled locally, while the other is offloaded to the MEC server for processing.

(1) Local Computation: Each user has a restricted computation capability for performing local computing, and the CPU frequency $f_{k}\left[n\right]$ serves as the key factor. The delay $T_{k}^{L}\left[n\right]$ and the energy consumption $E_{k}^{L}\left[n\right]$ for local computing can be, respectively, deduced as follows [32].

(3a) $$\begin{array}{cc} \; & T_{k}^{L}\left[n\right]=\frac{\beta_{k}\left[n\right]L_{k}\left[n\right]C_{k}}{f_{k}\left[n\right]} \end{array}$$

(3b) $$\begin{array}{cc} \; & E_{k}^{L}\left[n\right]=\eta T_{k}^{L}\left[n\right]f_{k}^{3}\left[n\right]=\eta \beta_{k}\left[n\right]C_{k}L_{k}\left[n\right]f_{k}^{2}\left[n\right] \end{array}$$

where $C_{k}$ is the number of CPU cycles required for computing, η denotes the effective capacitance coefficient for which $\eta f_{k}^{3}\left[n\right]$ is the CPU power consumption, $L_{k}\left[n\right]$ is the total task sizes, and $\beta_{k}\left[n\right]L_{k}\left[n\right]$ represents the task processed at the local level.

(2) Task Offloading: The offloading delay is determined by the offloading task size, which is given by

(4) $$\begin{array}{c} T_{k}^{o}\left[n\right]=\frac{\left(1-\beta_{k}\left[n\right]\right)L_{k}\left[n\right]}{R_{k}\left[n\right]} \end{array}$$

Similarly, the relevant transmission energy consumption of user task offloading is given by

(5) $$\begin{array}{c} E_{k}^{o}\left[n\right]=p_{u}T_{k}^{O}\left[n\right]=\frac{\left(1-\beta_{k}\left[n\right]\right)L_{k}\left[n\right]p_{u}}{R_{k}\left[n\right]} \end{array}$$

(3) UAV Computation: Once the user offloads the task to the MEC server, the UAV processes the task, which causes the processing delay as

(6) $$\begin{array}{c} T_{k}^{c}\left[n\right]=\frac{\left(1-\beta_{k}\left[n\right]\right)L_{k}\left[n\right]C_{k}}{f_{u,k}\left[n\right]} \end{array}$$

where $f_{u,k}\left[n\right]$ denotes the CPU computing frequency of the UAV allocated to user *k*.

Similar to (3b), the energy consumption for offloaded task processing can be obtained as

(7) $$\begin{array}{c} E_{k}^{c}\left[n\right]=\psi f_{u,k}^{3}\left[n\right]T_{k}^{c}\left[n\right]=\psi f_{u,k}^{2}\left[n\right]\left(1-\beta_{k}\left[n\right]\right)L_{k}\left[n\right]C_{k} \end{array}$$

where ψ is the UAV effective capacitance coefficient.

(4) Result Feedback: Once the UAV task computation is completed, the results will be fed back to the relevant user, and the transmission-induced delay $T_{k}^{u}\left[n\right]$ is given by

(8) $$\begin{array}{c} T_{k}^{u}\left[n\right]=\frac{L_{u}\left[n\right]}{R_{u}\left[n\right]} \end{array}$$

where $L_{u}\left[n\right]$ represents the transmission data size back to the user.

Then, the energy consumption for the information feedback is

(9) $$\begin{array}{c} E_{k}^{u}\left[n\right]=p_{u}T_{k}^{u}\left[n\right]=\frac{L_{u}\left[n\right]p_{u}}{R_{u}\left[n\right]} \end{array}$$

From (3) to (9), the total delay and energy consumption in each time slot are given by

(10a) $$\begin{array}{cc} \; & T\left[n\right]=\sum_{k=1}^{K}T_{k}^{L}\left[n\right]+T_{k}^{o}\left[n\right]+T_{k}^{c}\left[n\right]+T_{k}^{u}\left[n\right] \end{array}$$

(10b) $$\begin{array}{cc} \; & E\left[n\right]=\sum_{k=1}^{K}E_{k}^{L}\left[n\right]+E_{k}^{o}\left[n\right]+E_{k}^{c}\left[n\right]+E_{k}^{u}\left[n\right] \end{array}$$

### 3.3. UAV Control Model

The existing solutions for UAV trajectory planning are typically carried out under the assumption of perfect UAV flight capability, in which case the velocity and acceleration of UAV can change arbitrarily. However, it is impractical in actual UAV flight, considering the constraints on acceleration and velocity as well as the underlying dynamics principles. In addition, the time-varying task requirements of users and the dynamic transmission environment contribute to the frequent adjustments in the UAV trajectory. Therefore, real-time UAV trajectory control is raised to reduce the performance degradation induced by the state deviations.

Typically, the dynamics of the UAV can be expressed as

(11a) $$\begin{array}{c} \dot{q}\left(t\right)=v\left(t\right) \end{array}$$

(11b) $$\begin{array}{cc} \; & \dot{v}\left(t\right)=a\left(t-\Delta \right) \end{array}$$

where q(t) and v(t), respectively, denote the UAV’s location and velocity, a(t) is the UAV acceleration, and Δ is the time delay.

Define a new state vector as

(12) $$\begin{array}{c} w\left(t\right)=\left[q\left(t\right),\dot{q}\left(t\right)\right]=\left[q\left(t\right),v\left(t\right)\right)] \end{array}$$

Based on (11) and (12), the dynamics model can be rewritten as

(13) $$\begin{array}{c} \begin{array}{c} \dot{w}\left(t\right)=Aw\left(t\right)+Ba\left(t-\Delta \right) \end{array} \end{array}$$

where

$$A=\left[\left.\begin{array}{cccccc} 0 & 0 & 0 & 1 & 0 & 0\\ 0 & 0 & 0 & 0 & 1 & 0\\ 0 & 0 & 0 & 0 & 0 & 1\\ 0 & 0 & 0 & 0 & 0 & 0\\ 0 & 0 & 0 & 0 & 0 & 0\\ 0 & 0 & 0 & 0 & 0 & 0 \end{array}\right]\right.,\;B=\left[\left.\begin{array}{ccc} 0 & 0 & 0\\ 0 & 0 & 0\\ 0 & 0 & 0\\ 1 & 0 & 0\\ 0 & 1 & 0\\ 0 & 0 & 1 \end{array}\right]\right.$$

Then, the relevant discrete-time dynamics is given by [33]

(14) $$\begin{array}{c} w\left[n+1\right]=A_{0}w\left[n\right]+B_{1}a\left[n\right]+B_{2}a\left[n-1\right] \end{array}$$

where

(15) $$\begin{array}{c} A_{0}=e^{A\Delta T},\;B_{1}=\int_{0}^{\Delta T-\Delta }e^{A\Delta T}dtB,\;w\left[n\right]=w\left[n\Delta T\right],\;B_{2}=\int_{\Delta T-\Delta }^{\Delta T}e^{A\Delta T}dtB, \end{array}$$

where a[n] is the control strategy (i.e., acceleration) of UAV, and ΔT is the duration of one time slot.

The propulsion energy is the significant flight energy consumption for the UAV, which is typically given by [34]

(16) $$\begin{array}{cc} E_{u}^{fly}\left[n\right]= & \left(N_{1}{∥v[n]∥}^{3}+\frac{N_{2}}{∥v[n]∥}\left(1+\frac{{∥a[n]∥}^{2}}{a_{0}^{2}}\right)\right)\Delta T+\frac{1}{2}M({∥v\left[n\right]∥}^{2}-{∥v\left[n-1\right]∥}^{2}) \end{array}$$

where $N_{1}$ and $N_{2}$ are system-determined parameters, *M* is the mass of the UAV, and $a_{0}$ is the gravitational acceleration.

### 3.4. Optimization Problem Formulation

The objective of UAV-assisted MEC is to minimize the weighted energy consumption and delay through the optimization design of UAV trajectory, task offloading strategy, and the allocation of computation resources for both UAV and users, which can be formulated as

$$\begin{array}{c} P1:\min_{F,\beta ,Q}\sum_{n=1}^{N}\varepsilon_{1}E_{u}^{fly}+\varepsilon_{2}E\left[n\right]+\varepsilon_{3}T\left[n\right] \end{array}$$

(17a) $$\begin{array}{c} \;s.t.\;w\left[n+1\right]=A_{0}w\left[n\right]+B_{1}a\left[n\right]+B_{2}a\left[n-1\right] \end{array}$$

(17b) $$\begin{array}{c} \sum_{k=1}^{K}f_{u,k}\leq F_{u,\max},\;\forall k \end{array}$$

(17c) $$\begin{array}{c} \;0\leq f_{u,k},\;\forall k \end{array}$$

(17d) $$\begin{array}{c} \;0\leq f_{k}\leq F_{k},\;\forall k \end{array}$$

(17e) $$\begin{array}{c} \;0\leq \beta_{k}\leq 1,\;\forall k \end{array}$$

where $F\;≜\;\{f_{k}\left[n\right],f_{u,k}\left[n\right]\}$ represents the assigned CPU frequencies, $\beta \;=\;\{\beta_{k}\left[n\right]\}$ is the offloading ratio, $Q\;=\;\{q_{u}\left[n\right]\}$ is the UAV flight trajectory, and $\varepsilon_{1}$, $\varepsilon_{2}$, $\varepsilon_{3}$ are the weight coefficients. In the optimization problem, (17a) is the corresponding discrete-time dynamics of the UAV, (17b)–(17d) are the maximum CPU computation frequency constraints, and (17e) is the offloading ratio constraint of the user.

## 4. LLDDPG Algorithm Design

As mentioned above, P1 is a mixed optimization problem due to the system dynamics restriction (17a), which has been commonly ignored in existing studies. For a practical solution, the optimization problem P1 can be decomposed into three distinct subproblems. The detailed analysis will be presented below.

### 4.1. UAV Trajectory Design

Given the offloading ratio and the CPU computation frequency assignments, the optimization problem P1 can be simplified as a UAV trajectory design problem.

#### 4.1.1. DDPG-Based Desired Trajectory Design

Assuming the perfect UAV flight capacity, the optimization problem P1 can be simplified to a desired trajectory design challenge such that

(18) $$\begin{array}{c} P1.1:\min_{Q}\sum_{n=1}^{N}(\varepsilon E_{u}^{fly}+\sum_{k=1}^{K}\left(E_{k}^{o}\left[n\right]+E_{k}^{u}\left[n\right]\right)+\sum_{k=1}^{K}\left(T_{k}^{o}\left[n\right]+T_{k}^{u}\left[n\right]\right)) \end{array}$$

To address the subproblem of minimizing the system cost in terms of energy consumption and delay as in P1.1, deep reinforcement learning (DRL), which has shown remarkable ability in solving intricate network optimization challenges, is employed to achieve the desired trajectory of the UAV. Typically, DRL can be formulated as a Markov decision process (MDP), where the next system state depends solely on the current state and the action determined by the agent. To be specific, the MDP is defined by the tuple (S,A,R,P), where *S*, *A*, and *R*, respectively, represent the set of states, actions, and rewards, and *P* is the transition probability from state $s_{n}$ to state $s_{n+1}$ [35]. The following are the definitions for the state, action, and reward functions.

(1) State: Based on the MEC system model and task offloading model formulated in Section 3, the state $s_{n}$ consists of the locations of the UAV and users, as well as the user task requirements, which can be defined as

(19) $$\begin{array}{c} s_{n}=\left\{x_{1}\left[n\right],y_{1}\left[n\right],x_{2}\left[n\right],y_{2}\left[n\right],\cdots ,x_{K}\left[n\right],y_{K}\left[n\right],L_{1}\left[n\right],L_{2}\left[n\right],\cdots ,L_{K}\left[n\right],x_{u}\left[n\right],y_{u}\left[n\right]\right\} \end{array}$$

(2) Action: The UAV is required to determine its movements, including flight velocity v[n] and direction θ[n]), which is given by

(20) $$\begin{array}{c} a_{n}=\left\{v\left[n\right]\cos\left(\theta \left[n\right]\right),v\left[n\right]\sin\left(\theta \left[n\right]\right)\right\} \end{array}$$

(3) Reward: The reward function is highly associated with the optimization objective. The objective in P1.1 can be directly served as the reward function:

(21) $$\begin{array}{c} r_{n}=-\left(\varepsilon_{1}E_{u}^{fly}+\varepsilon_{2}\left(E_{k}^{o}+E_{k}^{u}\right)+\varepsilon_{3}\left(T_{k}^{o}+T_{k}^{u}\right)\right) \end{array}$$

In the proposed MDP framework, the UAV acts as the agent, interacting with the environment by observing a state $s_{n}$. Then, it executes an action $a_{n}$ based on its policy π. Following the execution of action $a_{n}$, the agent receives a reward $r_{n}$ and transitions to the next state $s_{n+1}$. DDPG, as one of the classic DRL algorithms, stands out for its ability to leverage low-dimensional observations to learn effective strategies in continuous action spaces. In dynamic environments, this method has been demonstrated to be highly effective in making decisions and achieving the desired trajectory of the UAV. Therefore, a DDPG-based algorithm for the design of desired UAV trajectory is proposed to minimize both the energy consumption and time delays induced by the UAV and users.

**Figure 2:**
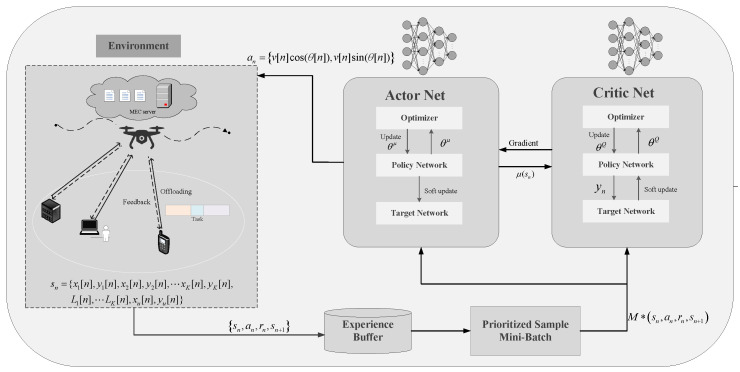
Schematic of DDPG algorithm.

The architecture of the DDPG-based algorithm is depicted in Figure 2. The DDPG network comprises actor and critic neural networks. Specifically, the actor network, serving as the policy network, takes the current environmental state as input and generates relevant actions through the analysis of the neural network. To enhance the efficacy of the iterative update strategy, the critic network, which utilizes a value-based learning approach, can be updated at each step.

The evaluation value $Q(s_{n},a_{n}|\theta^{Q})$ is acquired by executing action $a_{n}$ in the state $s_{n}$. The action $a_{n}=\mu \left(s_{n}|\theta^{\mu }\right)$ is taken in each state, reaching a specific value through a deterministic behavioral strategy. DDPG draws upon the dual network structure of DQN and experience replay to dissociate the behavior strategy network from the evaluation strategy network. The actor and critic have two networks with a similar structure but have asynchronous parameter renewals. In this manner, the convergence speed is quicker when training the network, and the soft update formula of the target network is set as follows:

(22a) $$\begin{array}{cc} \; & \theta^{Q^{\prime }}\leftarrow \tau \theta^{Q}+\left(1-\tau \right)\theta^{Q^{\prime }} \end{array}$$

(22b) $$\begin{array}{cc} \; & \theta^{\mu^{\prime }}\leftarrow \tau \theta^{\mu }+\left(1-\tau \right)\theta^{\mu^{\prime }} \end{array}$$

where τ represents the update rate, $\theta^{Q}$ and $\theta^{\mu }$ are the parameters of the critic network, while $\theta^{Q^{\prime }}$ and $\theta^{\mu^{\prime }}$ are the parameters of the target network.

The critic network parameter is updated as

(23) $$\begin{array}{cc} y_{i} & =R+\gamma Q^{\prime }\left(s_{n+1},\mu^{\prime }\left(s_{n+1}|\theta^{\mu^{\prime }}\right)|\theta^{Q^{\prime }}\right) \end{array}$$

where $y_{i}$ represents the actual evaluation value calculated by the target network, and γ is the reward decay rate.

Then, the loss function can be expressed as

(24) $$\begin{array}{c} L=\frac{1}{N}\sum_{n}(y_{n}-Q\left(s_{n},a_{n}|\theta^{Q}\right){)}^{2} \end{array}$$

The actor network parameter is updated by

(25) $$\begin{array}{c} \nabla_{\theta^{\mu }}J\approx \frac{1}{N}\sum_{i}\nabla_{a}Q\left(s_{n},a|\theta^{Q}\right) \end{array}$$

where $a\;=\;\mu \left(s_{n}\right)\nabla_{\theta^{\mu }}\mu \left(s_{n}|\theta^{Q}\right)$, ∇μ denotes the modification trend of the actor parameters, and ∇Q indicates the actor network update direction calculated by the critic.

After completing the training process, the optimized network parameters of the actor, denoted as $\theta^{\mu^{*}}$, are obtained. Subsequently, the desired UAV trajectory is given by

(26) $$\begin{array}{c} q^{*}\left[n\right]=\mu \left(s_{n}|\theta^{\mu^{*}}\right) \end{array}$$

Consequently, the DDPG-based algorithm for the design of desired UAV trajectory can be summarized in Algorithm 1.
**Algorithm 1** DDPG-based algorithm for desired UAV trajectory design**Input:** The positions of the UAV q(t) **Output:** UAV movement policy
1:Initialize the main actor network and critic network.2:Initialize the target actor network and critic network.3:Initialize the replay memory B and initialize $\sigma^{2}=2.0$, ε=0.9 for action exploration.4:**for** episode :=1,⋯,M **do**
5:   **for** step n:=1,⋯,N **do**
6:     Update the environment status, observe the current environment state $s_{n}$.
7:     Set the current action $a_{n}∼N\left(\mu \left(s_{n}|\theta^{\mu }\right),\in \sigma^{2}\right)$;8:     Execute the action $a_{n}$, obtain the reward $r_{n}$, and transit to the next state $s_{n+1}$.9:     Store the experience tuple ($s_{n}$, $a_{n}$, $r_{n}$, $s_{n+1}$) into replay memory B.10:     **if** Update **then**11:        Randomly sampling the mini-batch transitions from B.12:        Renew the critic network through minimizing the critic loss.13:        Renew the actor network through maximizing the actor loss.14:        Renew the target networks based on (29).15:        Decay the action $\sigma^{2}\leftarrow \sigma^{2}\varepsilon$16:     **end if**17:   **end for**18:**end for**

#### 4.1.2. LQR-Based Trajectory Tracking Control

In practical applications, the UAV faces a dynamic transmission environment, time-varying user task requirements, and diverse flight disturbances. To enhance system performance and control stability, the UAV must dynamically adjust its flight trajectory. In addition, due to the inherent limitations in the UAV’s velocity and acceleration, the actual flight state cannot be adjusted arbitrarily to match the desired trajectory $q^{*}$ obtained from subproblem P1.1. Therefore, there exist inevitable deviations between the desired and actual flight trajectories, potentially degrading the system performance and even significantly diminishing the effectiveness of the optimization design. To mitigate this issue, it is imperative to minimize the desired UAV trajectory deviation through real-time flight control. In this regard, an LQR-based UAV trajectory tracking control algorithm is proposed to align the actual flight trajectory with the desired trajectory as closely as possible, thereby improving the overall system performance.

Given the desired UAV trajectory design $q^{*}$ obtained from subproblem P1.1, the trajectory tracking control problem is given by

(27) $$\begin{array}{cc} P1.2: & q\to q^{*}\\ & s.t.\;w\left[n+1\right]=A_{0}w\left[n\right]+B_{1}a\left[n\right]+B_{2}a\left[n-1\right] \end{array}$$

The UAV location and velocity deviations can be, respectively, obtained as

(28a) $$\begin{array}{cc} \; & \tilde{q}=q-q^{*} \end{array}$$

(28b) $$\begin{array}{cc} \; & \tilde{v}=v-v^{*} \end{array}$$

Define a new vector $\tilde{w}=\left(\tilde{q},\tilde{v}\right)$, and then the UAV deviation dynamics can be derived based on (12), (13), and (14) as

(29) $$\begin{array}{c} \tilde{w}\left[n+1\right]=A_{0}\tilde{w}\left[n\right]+B_{1}a\left[n\right]+B_{2}a\left[n-1\right] \end{array}$$

Then, by using the typical quadradic cost funcion, the UAV trajectory tracking control problem P1.2 can be equivalent to be the following optimizaton problem [36]:

(30) $$\begin{array}{cc} \; & \min_{a[n]}{\tilde{w}}^{T}\left[n\right]Q\tilde{w}\left[n\right]+\sum_{n=0}^{N-1}{\tilde{w}}^{T}\left[n\right]Q\tilde{w}\left[n\right]+a^{T}\left[n\right]Ra\left[n\right]\\ \; & s.t.\;\tilde{w}\left[n+1\right]=A_{0}\tilde{w}\left[n\right]+B_{1}a\left[n\right]+B_{2}a\left[n-1\right] \end{array}$$

where *N* denotes the finite time horizon, and *Q* and *R* are system-determined parameters.

It can be seen that the objective of (30) is to minimize the trajectory tracking deviation through the optimal design of UAV flight a[n]. Then, an LQR-based trajectory tracking control algorithm is proposed to solve the optimization problem (30).

Define a new state vector as

(31) $$\begin{array}{c} z\left[n\right]=[\tilde{w}\left[n\right],a\left[n-1\right]] \end{array}$$

The optimization problem (30) can be rewritten as

(32) $$\begin{array}{cc} \; & \min_{a[n]}z^{T}\left[N\right]\dot{Q}z\left[N\right]+\sum_{n=0}^{N-1}z^{T}\left[n\right]\dot{Q}z\left[n\right]+a^{T}\left[n\right]Ra\left[n\right]\\ \; & \;s.t.\;z\left[n+1\right]=C_{n}z\left[n\right]+D_{n}a\left[n\right] \end{array}$$

where

(33) $$\dot{Q}=\left[\begin{array}{cc} Q & 0\\ 0 & 0 \end{array}\right],\;C\left[n\right]=\left[\begin{array}{cc} A[n] & B_{2}\left[n\right]\\ 0 & 0 \end{array}\right],\;D\left[n\right]=\left[\begin{array}{c} B_{1}\left[n\right]\\ 0 \end{array}\right]$$

The optimization problem (32) is a classic LQR-based control problem, and the optimal control strategy can be derived as [33]

(34) $$\begin{array}{c} a[n]=-l[n]z[n] \end{array}$$

where

(35) $$\begin{array}{c} l\left[n\right]={\left[D^{T}\left[n\right]S\left[n+1\right]D\left[n\right]+R\right]}^{-1}D^{T}\left[n\right]S\left[n+1\right]C\left[n\right],\\ S\left[n\right]=C^{T}\left[n\right]S\left[n+1\right]C\left[n\right]+\dot{Q}-l^{T}\left[n\right]D^{T}\left[n\right]S\left[n+1\right]C\left[n\right],\\ S\left[N\right]=\dot{Q} \end{array}$$

Based on (34) and (35), the actual UAV flight trajectory q[n] can be obtained based on the UAV acceleration strategy a[n].

### 4.2. Computation Resource Allocation Optimization

After the actual flight trajectory q[n] is determined from Section 4.1 and Section 4.2, the optimization problem in P1 can be equivalent to be a computation resource allocation problem, which is given by

(36a) $$\begin{array}{c} P1.3:\min_{F,\beta }\sum_{n=1}^{N}E_{k}^{L}\left[n\right]+E_{k}^{c}\left[n\right]+T_{k}^{L}\left[n\right]+T_{k}^{c}\left[n\right] \end{array}$$

(36b) $$\begin{array}{c} \;s.t.\;\sum_{k\in K}f_{k}\leq F_{k},\forall k \end{array}$$

(36c) $$\begin{array}{c} \;f_{k},f_{u}\geq 0,\forall k \end{array}$$

(36d) $$\begin{array}{c} \;0\leq \beta_{k}\leq 1,\forall k \end{array}$$

Subproblem P1.3, as a convex problem, can be typically solved using the Lagrange duality method as follows [37].

**Theorem** **1.** The UAV trajectory q[n], the CPU frequencies of the users and UAV, as well as the optimal offloading ratios, respectively, denoted by $\beta_{k}^{*}\left[n\right]$, $f_{u}^{*}\left[n\right]$, and $f_{k}^{*}\left[n\right]$, can be expressed as follows.

(37a) $$\begin{array}{cc} \; & f_{k}^{*}\left[n\right]=\sqrt{\frac{\gamma_{k}}{3\sigma_{c}MK\sum_{i=n}^{N}\kappa_{k,i}}},\;k\in K,n\in N \end{array}$$

(37b) $$\begin{array}{cc} \; & f_{u}^{*}\left[n\right]=\left\{\begin{array}{cc} 0, & n=1\\ \sqrt{\frac{\lambda_{N}-\sum_{i=n}^{N-1}\lambda_{i}}{3\sigma_{c}M}}, & n=2,\cdots ,N-1\\ \sqrt{\frac{\lambda_{N}}{3\sigma_{c}M}}, & n=N \end{array}\right. \end{array}$$

(37c) $$\begin{array}{c} \;\beta_{k}^{*}\left[n\right]=B\psi \log_{2}\left\{\frac{Bg_{k}\left[n\right]\left[\sum_{i=n+1}^{N-1}\lambda_{i}+\xi_{k}-\lambda_{N}\right]}{\sum_{i=n}^{N}\kappa_{k,i}\sigma^{2}\ln2}\right\} \end{array}$$

*where $\xi_{k}\geq 0$, $\kappa_{k,n}\geq 0$, $\lambda_{n}\geq 0$.*

**Figure 3:**
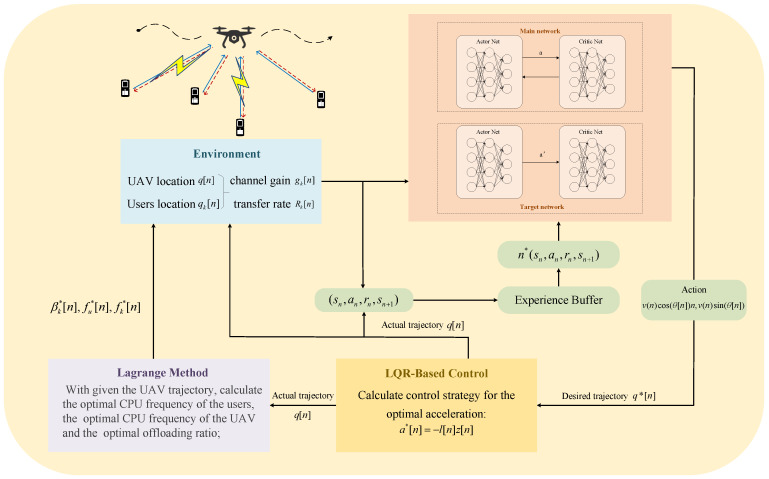
Schematic of the proposed LLDDPG algorithm.

Finally, based on the algorithm analysis presented in Section 4.1 and Section 4.2, the schematic of the proposed LLDDPG algorithm for the joint optimization of the UAV dynamic trajectory and resource allocation can be illustrated as shown in Figure 3, and its algorithmic procedure can be summarized in Algorithm 2.
**Algorithm 2** LLDDPG Algorithm1:Initialization: $q_{k}$, $q_{u}\left[0\right]$, $q_{u}\left[N\right]$, $L_{k}\left[0\right]$, $\beta_{k}\left[0\right]$, $f_{u,k}\left[0\right]$, $f_{k}\left[0\right]$, $p_{u}$, *p*, *B*, $C_{k}$, $N_{0}$, η, ψ.2:Solve the subproblem P1.1, and obtain the desired trajectory of UAV $q^{*}\left[n\right]$ based DDPG algorithm as Algorithm 1.3:Solve the subproblem P1.2 by using the LQR method:4:Derive the control coefficient l[n] offline based on (35).5:Obtain the acceleration control strategy a[n] as in (34) based on UAV state deviations and previous control strategies.6:Then, the actual UAV flight trajectory q[n] can be obtained based on acceleration control strategy a[n].7:Solve the subproblem P1.3 by using the Lagrange duality method:8:Obtain the optimal offloading ratios $\beta_{k}^{*}\left[n\right]$ and the CPU frequency of the users $f_{u}^{*}\left[n\right]$ and UAV $f_{k}^{*}\left[n\right]$ based on (37).9:q[n], $\beta_{k}^{*}\left[n\right]$, $f_{u}^{*}\left[n\right]$, and $f_{k}^{*}\left[n\right]$ are fed back for parameter update of DDPG algorithm.

## 5. Simulations

In this section, the performances of the proposed LLDDPG algorithm are comprehensively evaluated through simulations and numerical results. Specifically, the convergence performance of the algorithm is analyzed, and the performance comparisons with other existing works are given.

### 5.1. Simulation Settings

In simulations, the number of user devices is set to 100 and the task duration time is set to 10 min. The ground users are distributed in a 50 m × 50 m area. The flight height of the UAV is 10 m and the maximum flight velocity $V_{\max}$ = 10 m/s. At the beginning, the UAV starts the task at a random location. The vertical and horizontal coverage radius of the UAV are set, respectively, to $X_{d}$ = 25 m and $X_{h}$ = 10 m. The user’s data cache is updated each slot time with a Poisson process. The data buffer capacity $U_{\max}$ is set to 5000 packets, and the relevant data transfer size is *Q* = 10 Mbits. The UAV and user’s transmit power are, respectively, set to $P_{u}$ = 30 dBm and $P_{k}$ = −20 dBm. The other system corresponding parameters are shown in Table 1, where parameter settings refer to [38]. The structure and parameters of the DDPG network are shown in Table 2. During the implementation, the final output layer of the actor network is set to the tann layer, and all hidden layers are completely connected and activated using ReLU functions.

### 5.2. Results and Analysis

**Figure 4:**
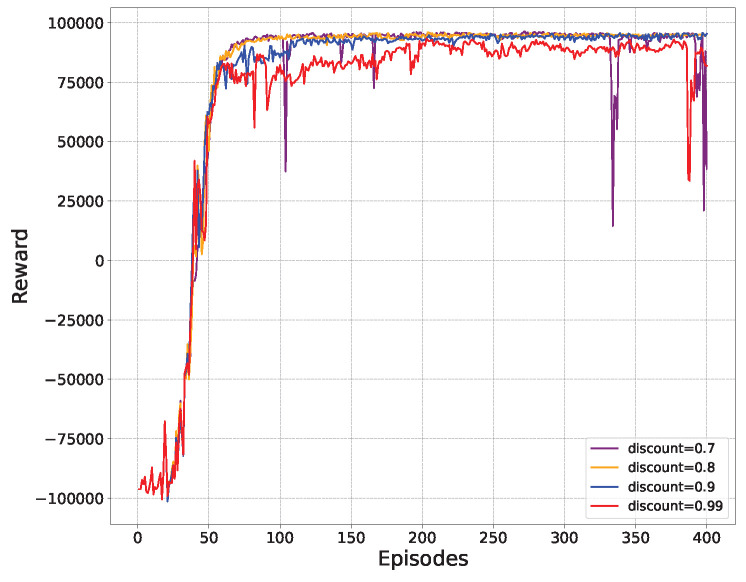
Reward of different discount factors.

Figure 4 shows the convergence of the reward function and the effect of different discount factors on the reward. The results reveal that the model convergence speed is relatively fast, and the final convergence level of the reward function is comparable under the conditions of different discount factors. When the discount factor is 0.99 and 0.7, there are abnormal fluctuations in the subsequent convergence stage, indicating that the exploration of action space is not comprehensive. Since there is no significant change in the final performance when the discount factor is 0.9, it indicates that the agent is able to learn the optimal policy.

**Figure 5:**
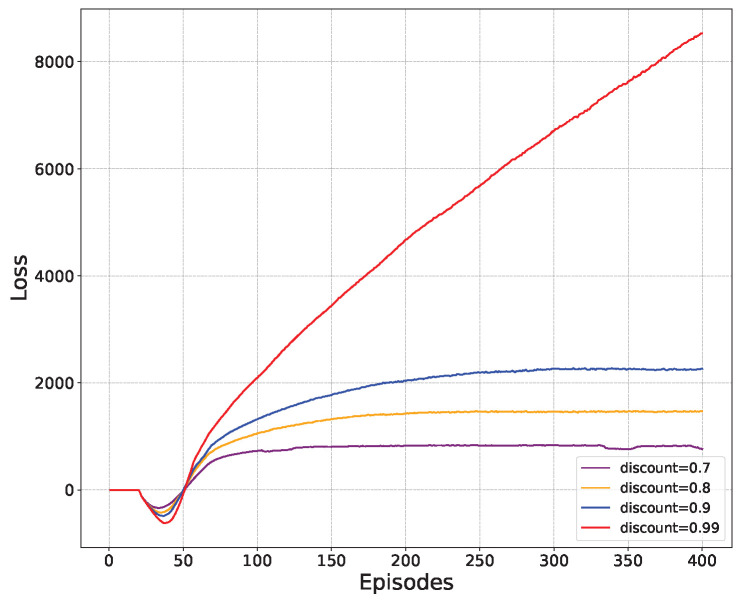
Loss of different discount factors.

Figure 5 presents the correlation between the learning rate and the loss function. Initially, the learning rate shows a high sensitivity to the loss function of the model. In the case of a low learning rate, the value of the loss function still increases slowly even after multiple training episodes. Conversely, in the case of a higher learning rate, the loss function rises rapidly, but it eventually takes a long time to converge. To ensure more comprehensive exploration of the agent’s action space, it is better to keep a smooth increase in the loss function and ultimately reach the optimal value. To sum up, the discount factor 0.9 is selected as a moderate discount factor to achieve the desired result.

**Figure 6:**
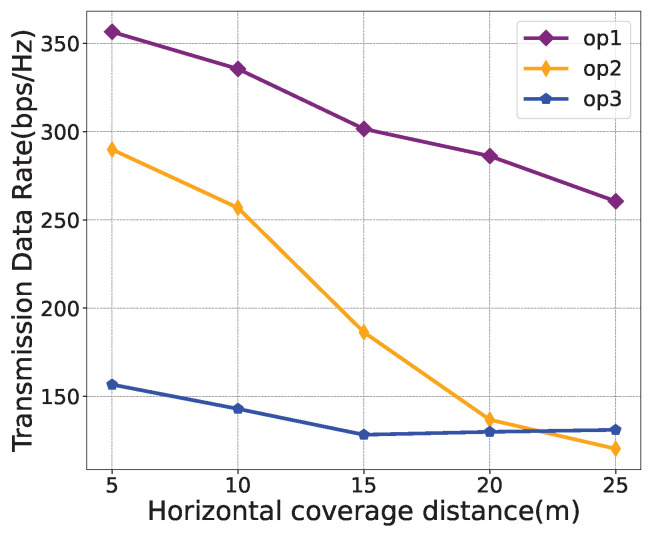
Transmission data rate comparison under different weight parameters.

**Figure 7:**
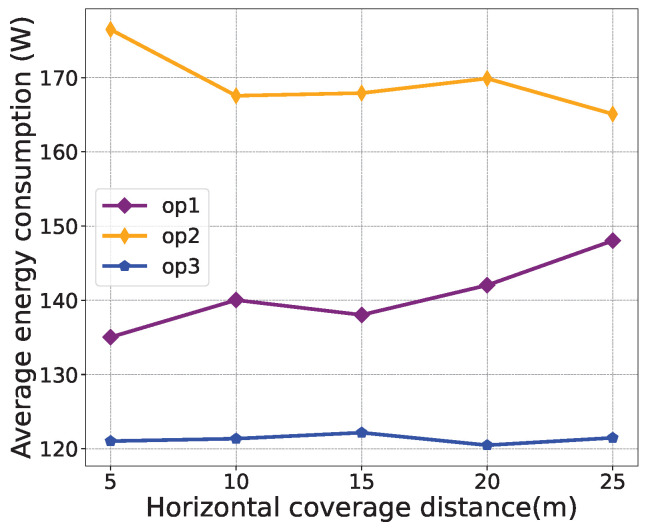
Average energy consumption comparison under different weight parameters.

**Figure 8:**
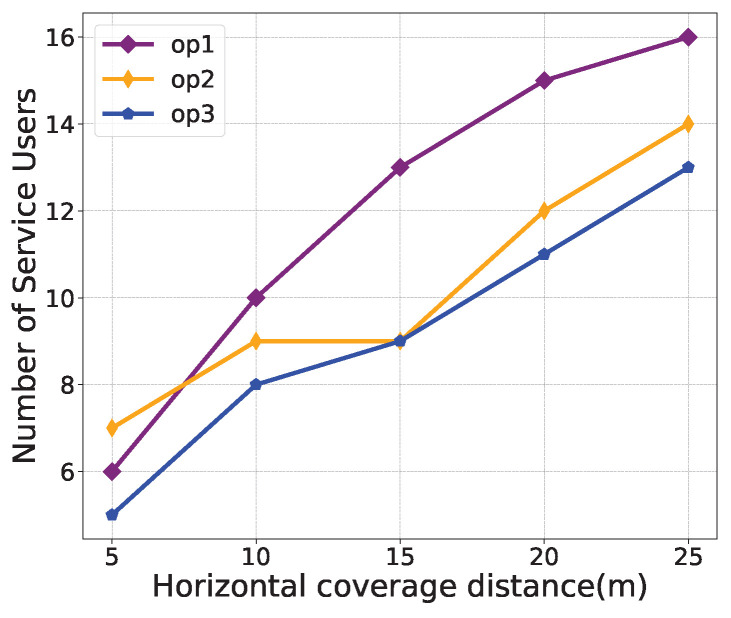
Number of service users comparison under different weight parameters.

For performance evaluations, Figure 6, Figure 7 and Figure 8 illustrate the effects of different weight parameters, namely $\varepsilon_{1}$, $\varepsilon_{2}$, and $\varepsilon_{3}$, on different system performances, including the transmission data rate, average energy consumption, and number of service users. The parameters for the comparison experiments are set as shown in Table 3. The horizontal coverage distance is set to 5 m, 10 m, 15 m, 20 m, and 25 m. It is observed from Figure 6 that, with the increase in the horizontal coverage distance, the transmission data rate decreases under all policies. In Figure 7, the average energy consumption under “op2” is higher than that of “op1” and “op3” because the weight of energy consumption is set to 0 in “op2”. As can be seen from Figure 8, as the horizontal coverage distance increases, the number of service users also increases. Overall, the transmission data rate and the number of service users under the “op1” policy are better than the other two strategies, and the energy consumption performance is slightly worse than that of the “op2” policy. This also validates that the proposed algorithm can successfully learn control strategies that simultaneously optimize multiple optimization objectives.

**Figure 9:**
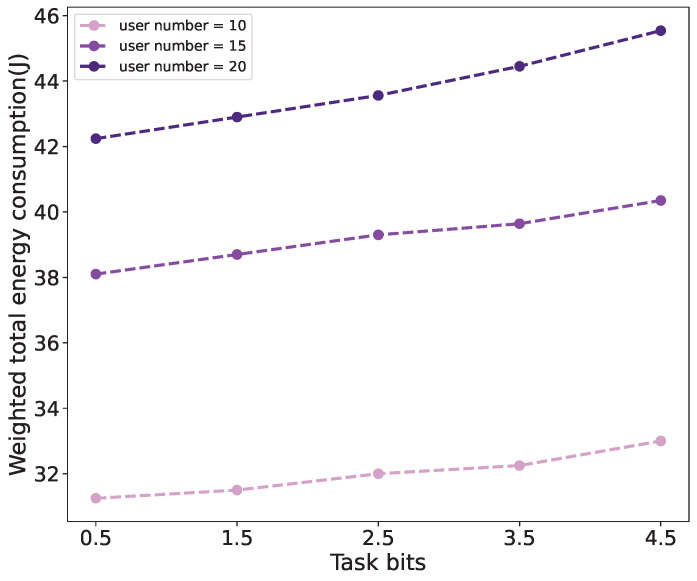
The relationship between weighted total energy consumption and tasks under different numbers of users.

Figure 9 shows the relationship between the weighted total energy consumption of all users and the quantity of user tasks under scenarios with different numbers of users. It can be seen that the weighted total energy consumption increases when the amount of computing tasks becomes larger, and this can be inferred from Formulas (3) and (5). In addition, the weighted total energy consumption increases with the increase in the number of users, owing to the requirement of consuming more energy between the UAV and the users for computing and transmission purposes.

**Figure 10:**
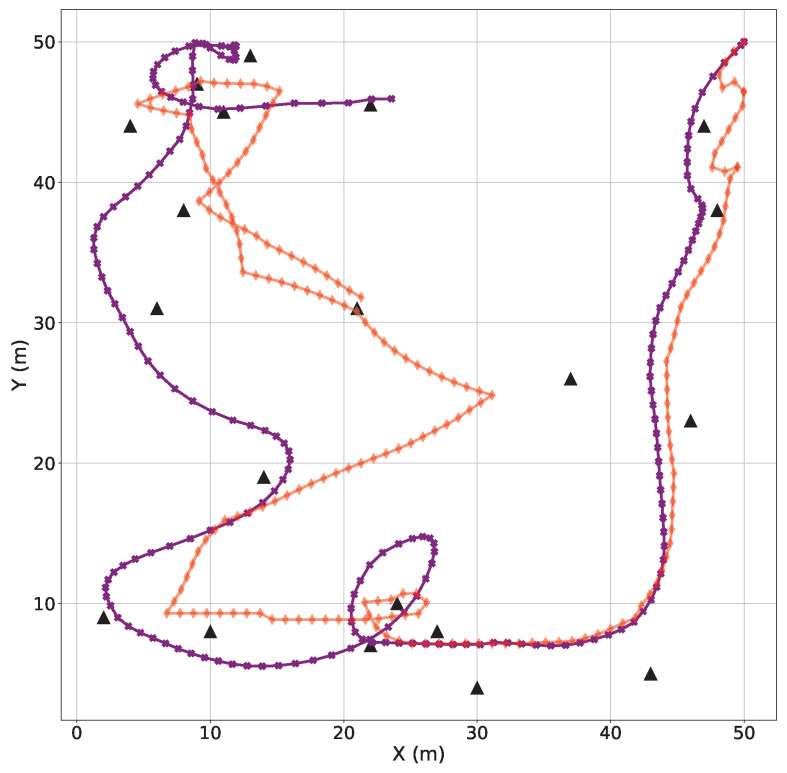
Comparisons between UAV desired and actual trajectories (actual: purple, desired: red).

**Figure 11:**
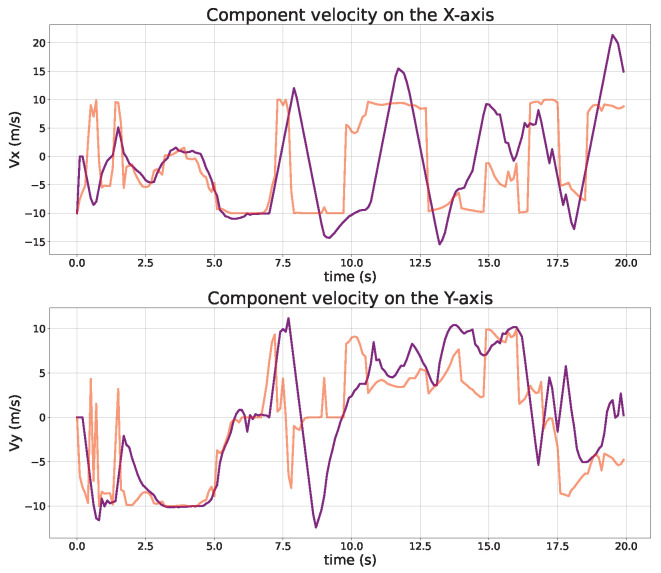
Comparisons of UAV velocity (actual: purple, desired: red).

**Figure 12:**
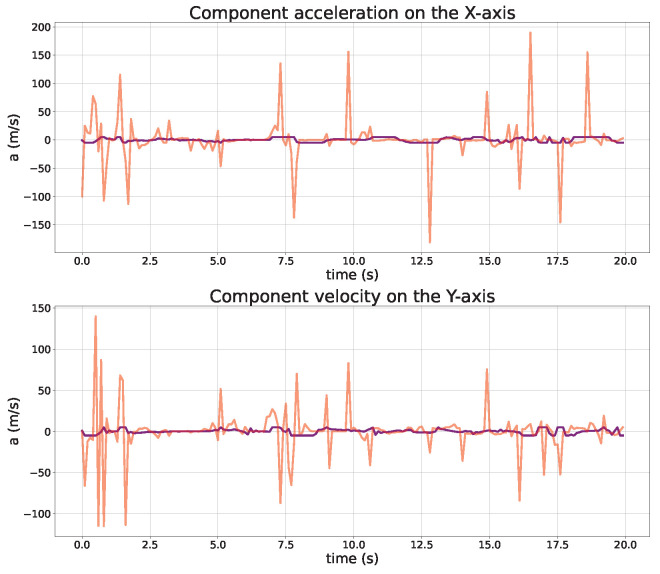
Comparisons of UAV acceleration (actual: purple, desired: red).

In order to investigate the effectiveness of the LLDDPG algorithm in actual UAV flight control, a numerical simulation of the scenario of UAV-assisted users in task offloading is conducted. The comparisons of the UAV trajectory, velocity, and acceleration are, respectively, presented in Figure 10, Figure 11 and Figure 12. It can be observed that the size of the offloading task for each user has a significant effect on the UAV trajectory design, and the UAV needs to approach the user with a higher task demand as quickly as possible. Initially, the global planning of the UAV trajectory is carried out based on the DDPG algorithm to ensure the performance of each user and save more energy consumption. Subsequently, the LQR algorithm is used to track the desired trajectory to mitigate the performance degradation introduced by the UAV dynamics constraints.

A rigorous trajectory analysis reveals that the initial alignment between the actual and desired trajectories is apparent. However, as the desired trajectory is formulated based on the user’s initial task size, it gradually diverges from the actual trajectory, which can be dynamically replanned in accordance with the evolving user task size and the current UAV flight state. Consequently, the deviation between the UAV’s actual and desired trajectories translates into an increasing gap. To address this, the proposed LLDDPG algorithm demonstrates its proficiency in dynamically adjusting the flight trajectory in real-time, taking into account both the user’s task requirements and the UAV’s current flight status, ultimately enhancing the overall performance.

**Figure 13:**
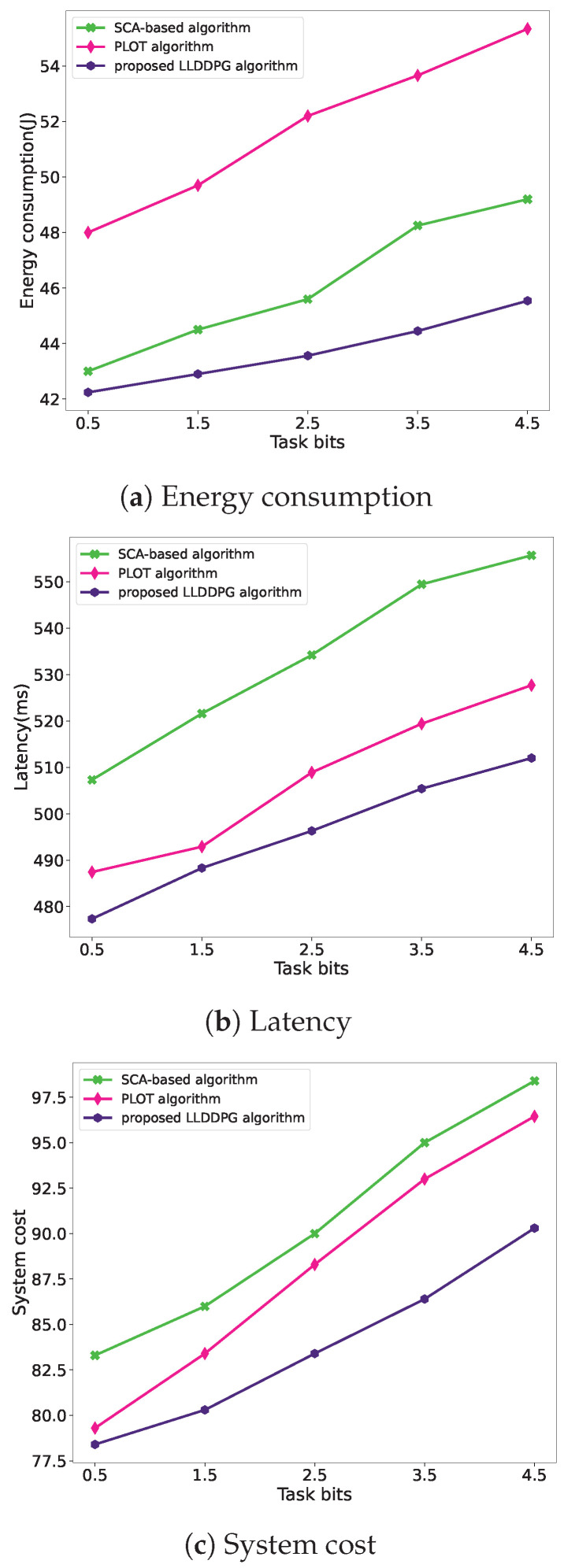
Comparison with other algorithms.

Figure 13 depicts the performance comparisons, including energy consumption, latency, and system cost, against existing optimization algorithms [24,25]. In [24], by leveraging the TSP with neighborhood and convex optimization techniques, an SCA-based algorithm is proposed to address the UAV trajectory planning problem, while the work in [25] proposed a PLOT control algorithm to maximize the aggregate execution of local and offloading tasks.

As depicted in Figure 13, it is revealed that, with the increasing task bit size, the energy consumption of the GCO algorithm escalates significantly greater than the others. Meanwhile, the TSP algorithm exhibits a notably higher latency compared to the other two algorithms. In contrast, the proposed LLDDPG algorithm achieves the lowest total system cost in the UAV-assisted MEC system, effectively optimizing the total weighted energy consumption and delay of the system.

## 6. Conclusions

This work focus on the intricate issue of resource allocation and real-time trajectory control for a UAV-assisted MEC system operating in a partial offloading mode. Through the joint optimization of CPU frequency, offload ratio, and UAV trajectory, the minimization of the weighted average energy consumption and delay is achieved. In particular, to address the trajectory planning problem, the DDPG and LQR algorithms are employed together to realize the real-time control for actual UAV flight. For the computation resource allocation problem, as a convex problem, a low-complexity Lagrange duality method is proposed to derive the optimal expressions for CPU frequency and offload ratio. Finally, the efficacy of the proposed LLDDPG algorithm is comprehensively evaluated through simulations and numerical results.

## Figures and Tables

**Table 1:** Simulation parameter settings.

Symbol	Description	Setting
*H*	UAV flight altitude	10 m
$V_{\max}$	Maximum flight velocity	10 m/s
*B*	Bandwidth	1 Mz
$\beta_{0}$	Channel power gain	−30 dB
$N_{0}$	Noise power	−90 dBm
a,b	LoS probability	10, 0.6
$C_{k}$	Number of CPU cycles per bit	1000 cycles/bit
ψ	Effective capacitance coefficient of UAV	$10^{-28}$
η	Effective capacitance coefficient of users	$10^{-28}$
τ	Time slot	0.1 s

**Table 2:** Parameters of DDPG training.

Parameter	Value
Learning rate of critic	0.001
Learning rate of actor	0.001
Update rate	0.001
Discount factor	0.9
Batch size	64

**Table 3:** Comparison of experiment parameters.

Name	Parameter
opt1	$\varepsilon_{1}=0.001$, $\varepsilon_{2}=0.01$, $\varepsilon_{3}=1$
op2	$\varepsilon_{1}=0$, $\varepsilon_{2}=0$, $\varepsilon_{3}=1$
op3	$\varepsilon_{1}=0$, $\varepsilon_{2}=1$, $\varepsilon_{3}=0$

## Data Availability

The original contributions presented in the study are included in the article; further inquiries can be directed to the corresponding author.
